# The Insect Chemoreceptor Superfamily in *Drosophila pseudoobscura*: Molecular Evolution of Ecologically-Relevant Genes Over 25 Million Years

**DOI:** 10.1673/031.009.1801

**Published:** 2009-05-08

**Authors:** Hugh M. Robertson

**Affiliations:** Department of Entomology, University of Illinois at Urbana-Champaign, Urbana, IL 61801, USA

**Keywords:** odorant receptor, gustatory receptor, smell, taste, *Drosophila melanogaster*

## Abstract

The insect chemoreceptor superfamily, consisting of the odorant receptor (Or) and gustatory receptor (Gr) families, exhibits patterns of evolution ranging from highly conserved proteins to lineage-specific gene subfamily expansions when compared across insect suborders and orders. Here their evolution across the timespan of 25 million years is examined which yield orthologous divergences ranging from 5–50%. They also reveal the beginnings of lineage-specific gene subfamilies as multiple duplications of particular gene lineages in either or both *Drosophila melanogaster* and *D. pseudoobscura* (Frolova and Astaurov) (Diptera: Drosophilidae). Gene losses and pseudogenes are similarly evident in both lineages, and even in closer comparisons of *D. melanogaster* with *D. yakuba*, leaving these species with roughly similar numbers of chemoreceptors despite considerable gene turnover. The large range of divergences and gene duplications provide abundant raw material for studies of structure and function in this novel superfamily, which contains proteins that evolved to bind specific ligands that mediate much of the ecology and mating behavior of insects.

## Introduction

The molecular basis of insect olfaction and gustation became amenable to study through discovery of two large families of genes that encode candidate chemoreceptor proteins in *Drosophila melanogaster* (Clyne et al. 1999; Vosshall et al. 1999; Clyne et al. 2000; Scott et. al. 2001; Dunipace et al. 2001; Robertson et al. 2003). These proteins have at least seven transmembrane domains and although once thought to be similar to the known G-protein-coupled receptor superfamilies, they share no sequence similarity with them (e.g. Hill et al. 2002) and appear to have the reverse membrane topology (Benton et al. 2006; Wistrand et al. 2006; Lundin et al. 2007). Genes encoding related receptors have been recognized in the genomes of other insects, including the mosquito *Anopheles gambiae* (Hill et al. 2002), the moths *Heliothis virescens* (Krieger et al. 2004) and *Bombyx mori* (Sakurai et al. 2004; Krieger et al. 2005; Wanner et al. 2007a), the red flour beetle *Tribolium castaneum* (Engsontia et al. 2008; Tribolium Genome Sequencing Consortium 2008), and the honey bee *Apis mellifera* (Robertson and Wanner 2006).

In *D. melanogaster*, the insect chemoreceptor gene superfamily consists of 60 odorant receptor (Or) and 60 gustatory receptor (Gr) genes encoding 62 and 68 different proteins through alternative splicing of long exons encoding the N-termini in some genes to one or more short exons encoding shared C-termini (Robertson et al. 2003). Examination of the molecular evolution of these genes indicates that the superfamily is old, at least as old as arthropods and perhaps older given the presence of five distantly related *gur* proteins encoded in the nematode *Caenorhabditis elegans* genome (unpublished results). There are many highly divergent gene lineages within the superfamily, with the Or family being a particularly highly expanded lineage, and these genes are now distributed throughout the *Drosophila* genome. In addition, signals of recent gene family evolution are apparent, including recent duplication of genes, either in tandem (e.g. Or22a/ b) or near each other (Or19a/b), polymorphism of pseudogenes (Or85e, Gr22b, and Gr22d are pseudogenes with single obvious defects in the sequenced Canton-S-based genome, while they are intact in the Oregon-R genome), and apparent movement of genes from tandem duplicated series to elsewhere in the genome (e.g. Gr5a and Gr61a from the Gr64a-f cluster) (Robertson et al. 2003).

Comparison of this *D. melanogaster* chemoreceptor repertoire with that encoded by the *Anopheles gambiae* mosquito genome sequence revealed that on this long timescale of approximately 250 Myr, there are few simple orthologous relationships, mostly involving a few highly conserved genes such as DmOr83b, Gr21a, and Gr63a (Hill et al. 2002). Both families reveal several complicated potentially orthologous relationships of one:many, many:one, and many:many genes, while the majority of the evolution involves differential gene subfamily lineage expansions and losses in these two highly divergent subordinal fly lineages. The availability of a draft genome sequence for another congeneric drosophilid fly, *D. pseudoobscura* (Frolova and Astaurov) (Diptera: Drosophilidae) (Richards et al. 2005), provides an opportunity to examine the patterns and processes of molecular evolution of these ecologically-relevant, and therefore presumably fairly rapidly evolving, genes on the timescale of approximately 25 Myr, or 10 times shorter than the *Drosophila: Anopheles* (Cyclorrhapha: Nematocera) subordinal comparison. An additional ten *Drosophila* species genomes have now also been sequenced (*Drosophila* 12 Genomes Consortium 2007). Additional comparisons with one of them, *D. yakuba*, which was the first to become available and represents a lineage roughly 10 Myr old from *D. melanogaster*, reinforces conclusions about evolutionary relationships of these genes.

## Materials and Methods

Gene models were built manually in the text editor of PAUP^*^v4.0b10 (Swofford 2002) using the output of TBLASTN searches of the 27 February 2003 *D. pseudoobscura* assembly available from the Baylor Human Genome Sequencing Center as guides. All DmOr and DmGr proteins described in Robertson et al. (2003) were employed as queries. These gene models were checked against the final draft assembly of 23 August 2003 (Freeze 2.0), as well as the unassembled reads in the Trace Archive at NCBI when necessary. Automated gene models were obtained from version 2.0 released from FlyBase in October 2005. The initial draft of the *D. yakuba* genome assembly was accessed from GenBank in August 2004, and gene models were updated using the DroYak2.1 assembly from the Washington University Genome Sequencing Center in August 2007.

Encoded proteins were aligned separately for each family using CLUSTALX (Jeanmougin et al. 1998) at default settings. For phylogenetic analysis the highly divergent N- and C-termini, that is, beyond the TM domains, and an internal segment corresponding to the long insertions in Or83b and Gr66a, respectively, were excluded. As a result, the low divergence of the most highly conserved proteins is slightly exaggerated in the trees, because these regions contain the few differing amino acids for some of them, e.g. Or83b and Gr21a. Corrected distances were calculated in TREE-PUZZLE v5.0 (Schmidt et al. 2002) using the BLOSUM62 amino acid matrix, and distance trees were estimated in PAUP^*^v4.0b10 using tree-bisection-and-reconnection branch swapping. Support for branches was obtained from 1000 bootstrap replications of uncorrected distance analysis using neighbor-joining.

## Results

### Gene model annotation difficulties

Draft genomes have sequence gaps between contigs that can disrupt gene models, and several such situations existed for the *D. pseudoobscura* assembly. Specifically, the DpOr genes were all intact, however an internal gap in DpGr2a and the C-termini of DpGr10b and 85a were obtained from unassembled reads that covered these gaps, although the DpGr10b model depends on a single low quality read. In the 2.0 assembly release, DpGr85a is now also separately assembled in the 4.5kb contig Unknown_group_751. Five DyOr and Gr genes were terminated by ends of contigs in the first genome release, however all are intact in the latest genome assembly.

For the Or and Gr families of roughly similar size in *D. pseudoobscura*., 18 and 8 proteins were precisely correctly annotated in FlyBase, respectively (Appendices 1 and 2), as part of the automated annotation effort (Richards et al. 2005). Existing annotations for 23 Ors and 24 Grs required some modification, commonly minor N- or C-terminal extensions to reach appropriate start and stop codons, but sometimes involving missed exons or unspliced open in-frame introns. In a few cases a single gene was annotated in a region that encodes multiple genes or transcripts, e.g. GA12528 represents the five transcripts that are hypothesized to be produced from the alternatively-spliced Gr28b locus. Eleven genes in each family were represented by GA placeholders in the *D. pseudoobscura* genome browser but no annotation was available for them, while 20 Ors and 16 Grs had no GA identifier associated with them. Some of the latter are pseudogenes and some were truncated by ends of contigs so they could not be annotated, and the remainder are instances of genes without *D. melanogaster* orthologs and hence might more easily have been missed by the orthology-based automated annotation process. The automated annotation rate was nevertheless rather low at just over 50%, perhaps because of the high divergences of some of these orthologous pairs. All of these gene models were communicated to FlyBase in 2005, and the encoded proteins are available in a supplementary file (chemoreceptor_proteins.txt).

Comparisons with the *D. pseudoobscura* and *D. yakuba* genes also allowed improvement of several *D. melanogaster* gene models, most of which were incorporated in FlyBase with publication of Robertson et al. (2003). Additional subsequent changes included recognition that the N-termini of DmGr21a and 63a are probably shorter than earlier predicted (see also Robertson and Kent 2009), while DmOr65b/c each likely have a short N-terminal extension to the existing annotation. These changes have been made in FlyBase. In addition, the version of DmOr98b in the sequenced genome is a pseudogene, see below.

### The Or family

**Figure 1.**
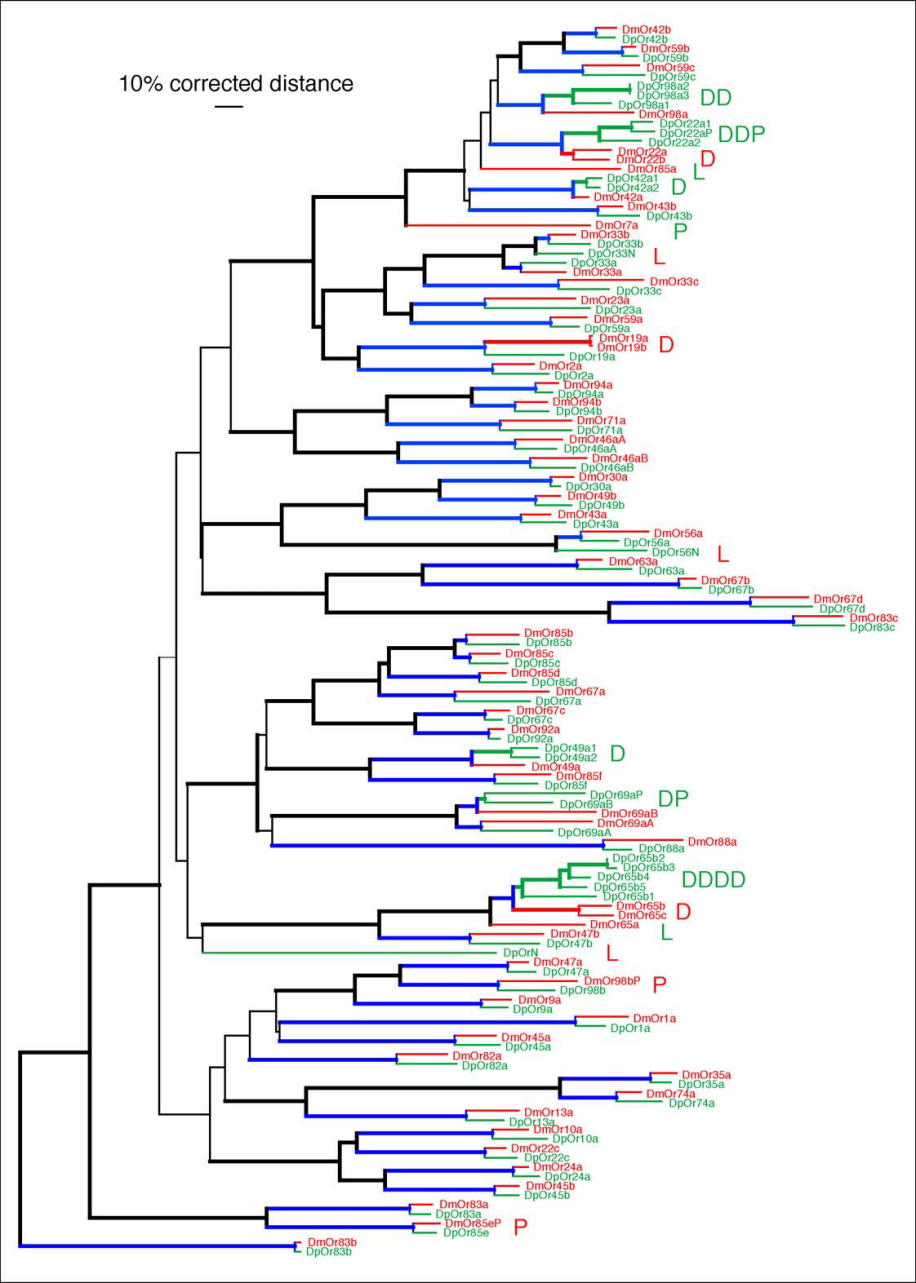
Phylogenetic relationships of the Or family in *Drosophila melanogaster* and *D. pseudoobscura*. This corrected distance tree was rooted by declaring the Or83b lineage as the outgroup, based on its position at the base of the Or family in analyses of the entire chemoreceptor superfamily (Robertson et al. 2003). DmOr proteins are shown in red and DpOr proteins in green. Proposed orthologous relationships, both simple and complicated, are indicated by blue branches connecting the Dm and Dp proteins. Letters on the right of the protein names highlight significant gene family evolutionary events: D — gene duplication; L — gene loss; P — pseudogene. DpOr7a is a highly degenerate pseudogene ortholog of DmOr7a and was not included in the tree analysis. Heavier lines indicate branches supported by at least 70% of 1000 bootstrap replications of uncorrected distance analysis. The terminal branches for Or33a were redrawn based on analyses of reduced datasets that included the *D. yakuba* proteins. Removal of extreme N- and C-termini plus an internal region of great length differences from the alignment for the phylogenetic analysis causes the most similar proteins to appear even more similar in this tree than they really are.

Comparisons of the *D. melanogaster* and *D. pseudoobscura* Or genes are shown in Appendix 1 and Figure 1. Identification of orthologous relationships was based on a combination of reciprocal best BLASTP matches, simple sister relationships in phylogenetic analysis (Figure 1), and examination of microsynteny with neighboring loci. In the Or family, there are 49 simple orthologs, with amino acid identities ranging from 94% for the highly conserved Or83b protein, which is known to be highly conserved throughout the endopterygote insects (Krieger et al. 2003), to around 50% for several relationships (e.g. Or19a/b, 23a, 65b/c, 67a, and 69aA/B). Most of the Ors are approximately twice as divergent as the average orthologous comparison for the entire proteome (Richards et al. 2005), confirming that these genes/proteins are among the more rapidly evolving portion of the genome.

Processes of gene family evolution that led to the major subfamily expansions and losses seen in the comparison with *A. gambiae* are evident on a smaller scale in the comparison with *D. pseudoobscura*. In addition to the recent duplication of the DmOr19a/b lineage, the comparison with *D. pseudoobscura* reveals that the DmOr22a/b duplication is younger than this species split and therefore specific to the *D. melanogaster* lineage. Remarkably, the orthologous locus in *D. pseudoobscura* has undergone two independent duplications leading to two apparently functional genes and a pseudogene. This comparison also reveals that DmOr65b/c were duplicated since the species split, while the orthologous locus in *D. pseudoobscura* has also seen repeated duplications yielding five apparently functional genes, and a N-terminal fragment not included in Figure 1 or Appendix 1. In addition there are two duplications and one triplication of other loci (Or42a, 49a, 98a) in the *D. pseudoobscura* lineage.

Balancing these gene duplications are several gene losses, including the orthologs of DmOr65a and 85a from *D. pseudoobscura*., while the ortholog of DmOr7a has been reduced to a fragmentary pseudogene. Reciprocally, *D. pseudoobscura* has three genes that appear to have been lost from *D. melanogaster* (DpOr33N, Or56N, and OrN). The first two of these are neighbors of genes with clear orthologs in *D. melanogaster*, and hence are given names reflecting these relationships, while the last one is a highly divergent lineage with no clear relationship to any DmOr. *D. melanogaster* also does not have an alternatively spliced exon in the Or69a locus that is a pseudogene in *D. pseudoobscura* (DpOr69aP). Examination of *D. yakuba* reveals that all three of these DpOr genes were lost before the *D. yakuba-D. melanogaster* split, but the DpOr69aP exon is intact in *D. yakuba*, so it was relatively recently lost from *D. melanogaster* and also became defective in *D. pseudoobscura*.

Comparison with *D. pseudoobscura*, as well as the *D. yakuba* and *D. simulons* draft genome assemblies, also led to recognition that DmOr98b is a polymorphic pseudogenic allele. A single base deletion in the sequenced genome causes a frameshift near the end of the second exon. Amplification of this region from genomic DNA of pooled animals of the New Jersey and Ives strains (for unknown reasons it would not amplify from the Oregon-R strain used previously to examine polymorphic Or and Gr pseudogenes), revealed that the New Jersey strain is also fixed for this single base deletion, but that the Ives strain is polymorphic. Amplifications from single Ives strain animals revealed six homozygotes for the deletion, three homozygotes for an intact allele, and three heterozygotes. The intact allele encodes the 20aa sequence MLISYQRTGELQPKFPFPSV at the end of exon 2, with the deletion removing an adenine in the third codon position of the first glutamine. Examination of the original traces from the Celera Whole Genome Shotgun *D. melanogaster* genome project reveals that this gene was polymorphic even within this strain, with 2 of 17 reads crossing the region having the intact allele.

In addition to these polymorphic and fragmentary pseudogenes in each genome, there are fragmentary pseudogene copies of the Or98a locus in each genome, however they do not appear to represent orthologous duplicates as they are not microsyntenic with each other (these are not shown in Appendix 1 or Figure 1). Thus there has been additional gene degeneration in each fly lineage leaving only fragments that will presumably eventually be lost completely from these genomes. Remarkably, a potential ortholog of one of these fragments remains intact in *D. yakuba*.

### The Gr family

**Figure 2.**
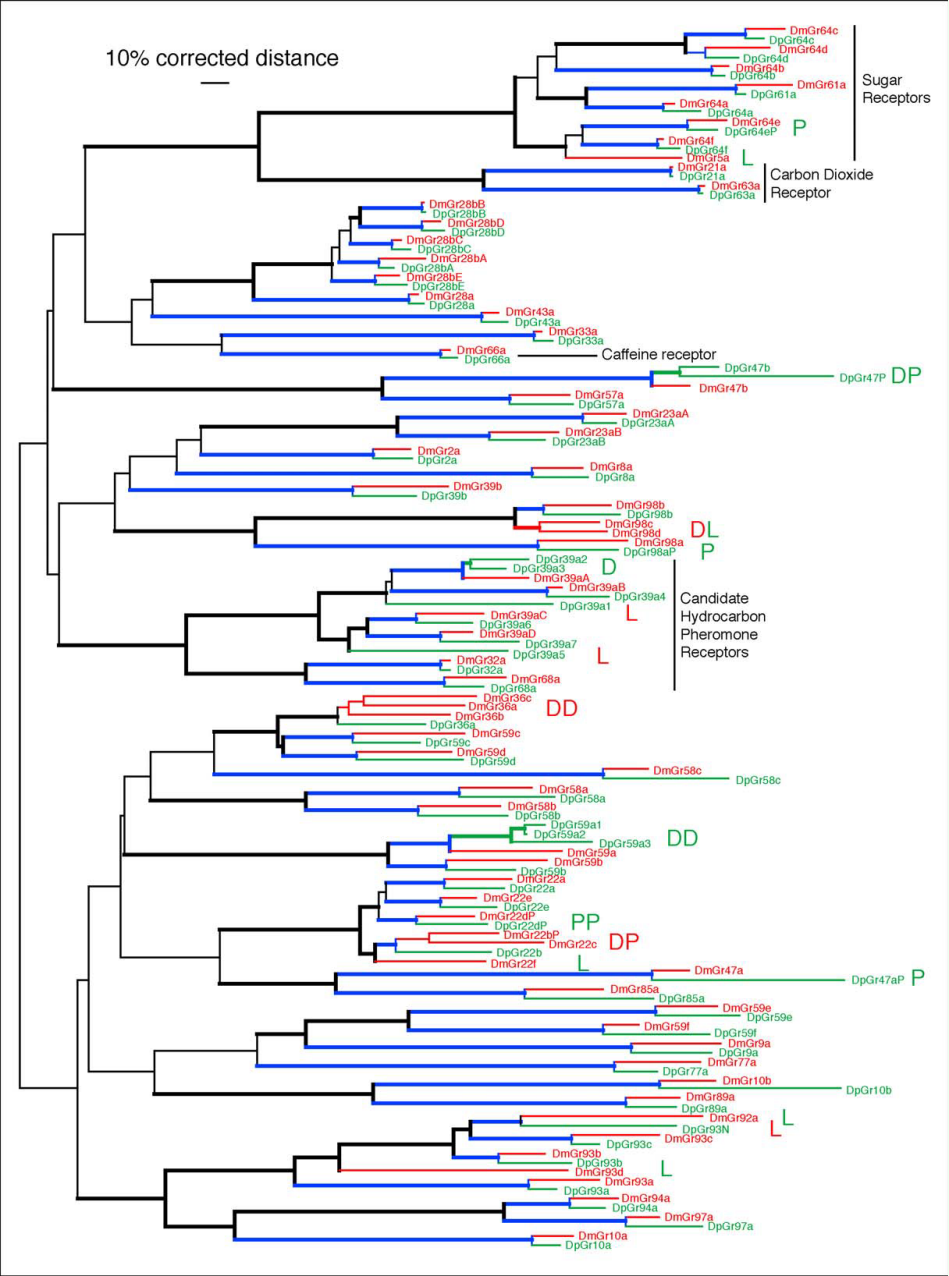
Phylogenetic relationships of the Gr family in *Drosophila melonogaster* and *D. pseudoobscura*. This corrected distance tree was rooted at the midpoint in the absence of a simple outgroup. Figure conventions are as in Figure 1 legend. Terminal branches were redrawn for Gr22b/c/f, 36a/b/c, 47b, and 64c/d. DpGr47P, 47aP, and 10b have extra long branches because in the first two instances parts of these proteins are missing, and in the latter, parts were reconstructed from a single read of poor quality.

The Gr family contains most of the protein diversity in the insect chemoreceptor superfamily (Robertson et al. 2003), from which the Ors are in reality a single highly expanded lineage. By most protein family criteria, the Gr family would be split into several families, including the highly divergent DmGr21a/63a lineage which form a heterodimeric olfactory receptor for carbon dioxide (Jones et al. 2007; Kwon et al. 2007) and a lineage of candidate sugar receptors related to the trehalose receptor DmGr5a (Chyb et al. 2003; Jiao et al. 2007; Slone et al. 2007; Dahanukar et al. 2007) (see top of Figure 2). The deeper divergences within the Gr family are also reflected in the lack of bootstrap support for most basal relationships (thin branches in Figure 2, versus Figure 1).

Analysis of the Gr family revealed patterns of evolution similar to the Ors (Appendix 2 and Figure 2). There are 54 pairs of apparently simple 1:1 orthologs. Their amino acid identities cover a similar range to the Ors, from 96% for the perfectly colinear Gr21a proteins to around 50% and multiple length differences for several proteins like Gr9a, Gr85a, and the Gr58 and Gr59 sets. The identification of Gr21a and 63a as a heterodimeric receptor for carbon dioxide (Jones et al. 2007; Kwon et al. 2007), which is presumably a very difficult molecule for proteins to interact with, might explain why so few amino acid substitutions are allowable that maintain function. Functional studies will be necessary to determine whether rapidly evolving receptors with around 50% amino acid identity like the Gr58 and Gr59 sets still detect the same ligands in both species - one can envisage that amino acid divergences in the range of 50% along with multiple length differences might indicate that the ligand specificity of these apparently orthologous but rapidly evolving receptors has changed.

There are several examples of gene birth in the Gr family in each species lineage, specifically DmGr22b/c, DmGr36a/b/c, and DmGr98c/d, while in *D. pseudoobscura* there are two duplications (Gr47bl/2 and Gr39a2/3) and one triplication (Gr59a1/2/3). Not all of these are simple examples however, primarily because some of these duplications are old and hence it is not clear whether in fact an ortholog has been lost from the other species instead. In the DmGr22a-f region, the DmGr22f gene I treated as having been lost from *D. pseudoobscura*, but some phylogenetic analyses suggest it is duplicated in the *D. melanogaster* lineage. The DpGr59a1–3 triplication is also complicated, with microsynteny analysis suggesting that this locus has undergone multiple duplications and gene losses in each lineage so that the simple “orthologous” comparisons to DmGr59a used in Appendix 2 might not be appropriate.

These Gr gene duplications are again balanced by gene losses, including the orthologs of DmGr5a, 22f, 92a, 93d, and 98c/d from *D. pseudoobscura*, while the orthologs of DpGr39a1 and a5, and of DpGr93N have been lost from *D. melanogaster*. In the case of DmGr5a, which has been shown to be a receptor for trehalose (e.g. Chyb et al. 2003), this means that *D. pseudoobscura* has either lost the ability to detect this sugar, or that this ability has been replaced with function of another receptor (see also Dahanukar et al. 2007). Comparisons with *D. yakuba* helped resolve several phylogenetic relationships, but also demonstrated that even more gene loss has occurred in this family, because it has four Grs which have been lost at least from *D. melanogaster* and sometimes also *D. pseudoobscura* (three of these are in the already complicated Gr59, 93, and 98 lineages, and the fourth is related to Gr85a).

While there are two Gr pseudogenes in the sequenced *D. melanogaster* strain (Gr22b and d), both of which are polymorphic in the species (Robertson et al. 2003), there are four pseudogenes in the sequenced *D. pseudoobscura* strain (Gr22d, 47a, 64e, and 98a). Remarkably Gr22d has independently become a pseudogene in each species. DpGr47a is a fragmentary pseudogene, but the lesions in the other three genes are single defects, so like Gr22b/d in *D. melanogaster*, these three pseudogenes in *D. pseudoobscura* might be polymorphic in the species. Indeed DpGr98a might not even be a pseudogene if the real start codon is considerably internal to that alignable with DmGr98a. There is also a recently duplicated pseudogene copy of DpGr47b that is truncated for the C-terminus.

### Gene movement

Richards et al. (2005) noted that while there were a large number of chromosomal rearrangements breaking up syntenic blocks of genes between these two species, the vast majority were intra-chromosome arm events, with few examples of translocations or transpositions between chromosome arms. This is also clearly evident from the locations of the *D. pseudoobscura* Or and Gr genes in Appendices 1 and 2. For example, in the entire Gr family every gene is still on the equivalent chromosome arm or Muller element (see Richards et al. 2005 for details of Muller elements), albeit usually changed in location along the arm. This means that the names of the genes in *D. melanogaster*, which indicate their chromosomal location, have little meaning in *D. pseudoobscura*, but I have chosen to use the same names for the orthologous *D. pseudoobscura* genes because most interest in these *D. pseudoobscura* genes derives from their comparison with the *D. melanogaster* genes and proteins.

The Or family has several examples of inter-chromosomal movement, however. Thus DpOr13a is on chromosome 4 instead of its expected location on XL; this appears to have been a retrotransposition mediated by reverse transcription of a mRNA, because DpOr13a is intronless and positioned between two genes whose orthologs in *D. melanogaster* are in 35A (left end of synteny block 626 in the Dp genome browser at FlyBase). Other examples of Or gene movement between chromosome arms are Or67a and Or92a, and the Or65b and Or98a expansions in *D. pseudoobscura*. None of these appear to involve retrotransposition because both *D. melanogaster* and *D. pseudoobscura* genes share introns. The direction of transposition cannot be determined by microsynteny analysis alone for Or67a and Or92a. For Or67a this single gene in synteny block 190 moved; for Or92a the *D. pseudoobscura* gene is 30kb in from the end of 12.5Mbp scaffold XL_le with no neighboring orthologs so nothing can be said about it. In the case of the DpOr65b and DpOr98a expansions in *D. pseudoobscura*, it appears that these transpositions occurred in the *D. pseudoobscura* lineage, perhaps concomitantly with the duplications of these genes, because in each case there is one copy of the DpOr that is microsyntenic with the single DmOr ortholog.

## Discussion

The overall pattern of evolution of these chemoreceptor genes in the recent *Drosophila* lineage appears to be a balance of birth/duplication and death/loss of genes. Thus 25 have become pseudogenes or were lost in the lineages leading to *D. melanogaster* and *D. pseudoobscura* (Table 1), although some pseudogenes are polymorphic, while 22 have been born through gene duplication, leaving each species with roughly the same number of encoded proteins. This stability of total functional Or number despite considerable gene turnover was recently reported by Nozawa and Nei (2007) and Guo and Kim (2007) for comparisons across most of the 12 available *Drosophila* species, although the Hawaiian *D. grimshawi* appears to have a relative expansion of this family. Remarkably, however, both the Ors and Grs show great acceleration of pseudogenization in the specialist species *D. sechellia*, which is a sibling of the generalist *D. simulans*, revealing how an ecological revolution can rapidly change the evolutionary dynamics of these chemoreceptors (Dekker et al. 2006; McBride 2007).

**Table 1.**
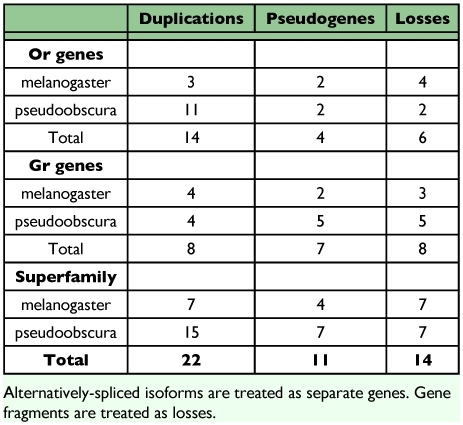
Gene duplications, pseudogenizations, and losses in Drosophila *melanogaster* and *D. pseudoobscura*.

Comparisons of genes and their encoded proteins between *D. melanogaster* and *D. pseudoobscura* proved to be too distant to provide much useful information on the patterns of selection pressures (Richards et al. 2005). Simple analyses of Ka/Ks ratios of non-synonymous to synonymous changes reveal that all of these chemoreceptor genes are under strong purifying selection pressure across this 10–25 Myr timescale when considered across their entire lengths (results not shown). This is in agreement with the results of Tunstall et al. (2007) for 10 Ors and one Gr considered from 8–12 species between *D. melanogaster* and *D. pseudoobscura*. Their generation of additional sequences for each chemoreceptor lineage allowed them to apply more sophisticated tree-based maximum likelihood methods that yielded suggestions of positive selection on three genes in particular lineages, or on 12 codons within four genes. Guo and Kim (2007) extended these kinds of analyses to the entire Or family across all 12 species with genome sequences, however they found signals of positive selection in a different set of genes than Tunstall et al. (2007). More detailed analyses of the entire superfamily, but limited to the *D. melanogaster* species group, provide additional insights into patterns of selection on these genes (McBride et al. 2007).

This comparison across the past 25 Myr provides a window into the processes by which these two large, ecologically-relevant gene families have evolved over much longer timescales, leading to the largely species-specific gene lineages seen in comparisons with mosquitoes (Hill et al. 2002), moths (Wanner et al. 2007a), beetles (Engsontia et al. 2008; Tribolium Genome Sequencing Consortium 2008), and bees (Robertson and Wanner 2006). The lineages that are still orthologous in these distant comparisons, e.g. the DmOr83b “chaperone” (Krieger et al. 2003; Larsson et al. 2004; Neuhaus et al. 2005) and the DmGr21a/63a carbon dioxide heterodimer (Jones et al. 2007; Kwon et al. 2007; Lu et al. 2007; Robertson and Kent 2009) are the most highly conserved lineages within these *Drosophila* species. Another set of relatively well-conserved proteins are the candidate sugar receptors related to the trehalose receptor DmGr5a (Chyb et al. 2003; Jiao et al. 2007; Slone et al. 2007; Dahanukar et al. 2007), although remarkably that particular gene was lost from the *D. pseudoobscura* lineage. The only other lineages showing simple orthology out to the honey bee are the DmGr43a lineage of unknown ligand specificity and the alternatively spliced DmGr28a/b loci (Robertson and Wanner 2006), both of which are highly conserved between *D. melanogaster* and *D. pseudoobscura*. The DmGr28a/b proteins have recently been shown to be expressed in both chemosensory neurons and other neurons not obviously involved in gustation (Thorne and Amrein 2008). In stark contrast, several lineages in each gene family and sometimes each species, e.g. Or22, 65, 98a and Gr22, 36, 39, 59a, 98a, show lineage-specific expansions. Combined with the relatively high rate of gene loss, it is not hard to see how most of these insect chemoreceptors come to be quite different in more distant comparisons across orders of insects.

**Figure 3.**
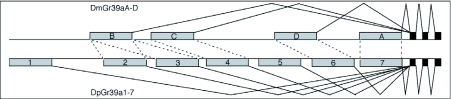
Schematic diagram of the Gr39a loci that are hypothesized to be alternatively-spliced. Grey boxes indicate the long alternatively-spliced N-terminal exons, while the three black boxes indicate the shared C-terminal exons. Two upstream exons present in *pseudoobscura* (1 and 5) were lost from *Drosophila melanogoster* (and *D. yakuba*), while one was duplicated in *D. pseudoobscura* (2/3).

Examination of several other receptors of particular interest reveals various levels of conservation. DmOr67d, a gene model contributed by Robertson et al. (2003), has been shown to be a receptor for cis-vaccenyl acetate, a volatile chemical that serves as an aggregation, male sex, and mating deterrent pheromone (Ha and Smith 2006; van der Goes van Naters and Carlson 2007; Kurtovic et al. 2007; Ejima et al. 2007). Or67d is not particularly highly conserved, at 64% identity, so it will be of interest to determine whether the DpOr67d receptor also recognizes this ligand. Two other chemoreceptors of interest in the context of mating behavior are the sister DmGr68a and 32a proteins implicated in recognition of female-specific cuticular hydrocarbons by males during tapping and licking of the female, although their specific ligands have not been identified (Bray and Amrein 2003; Ebbs and Amrein 2007). Gr68a is not particularly highly conserved at 67% amino acid identity, however Gr32a is amongst the most well conserved at 85% identity. Perhaps even more interesting is the implication that the related Gr39a set of protein isoforms (Figures 2) might also be involved in pheromone perception (Ebbs and Amrein 2007). In addition to high divergence between the four orthologous proteins in this set (62–75% identity - Appendix 2 and Figure 2), this protein set has undergone considerable lineage-specific evolution, with two losses in the common ancestor of *D. melanogaster* and *D. yakuba*, and a duplication in *D. pseudoobscura* (Figure 3).

Many of the remaining Grs are likely to be bitter taste receptors and DmGr66a has been shown to detect caffeine (Thorne et al. 2004; Wang et al. 2004; Marella et al. 2006; Ebbs and Amrein 2007). Again this is a relatively highly conserved protein with 84% identity so is likely to have the same ligand specificity in other *Drosophila* species. Most DmOr ligand specificities have now been established (e.g. Hallem and Carlson 2006), however there are few obvious relationships between the level of conservation of the receptors and the characteristics of their ligands. A possible pattern beyond their ligands is that receptors involved in formation of heterodimers, that is Or83b, Gr21a, Gr63a, and Gr66a, show higher conservation than most others, presumably reflecting in part the need to maintain many residues to sustain dimerization with other receptors. In addition to misexpression of olfactory receptors in particular empty olfactory sensory neurons followed by single-sensillum recordings (e.g. Dobritsa et al. 2003; Hallem and Carlson 2006; Jones et al. 2007; Kwon et al. 2007), several different methods for studying ligand specificity in heterologous expression systems are now available (e.g. Chyb et al. 2003; Neuhaus et al. 2005; Kiely et al. 2007; Wanner et al. 2007b). The varying levels of amino acid divergence in related *Drosophila* species might provide a useful resource for studies of structure and function in this novel superfamily of proteins using both endogenous and heterologous expression systems.

## Figures and Tables

**Table:**
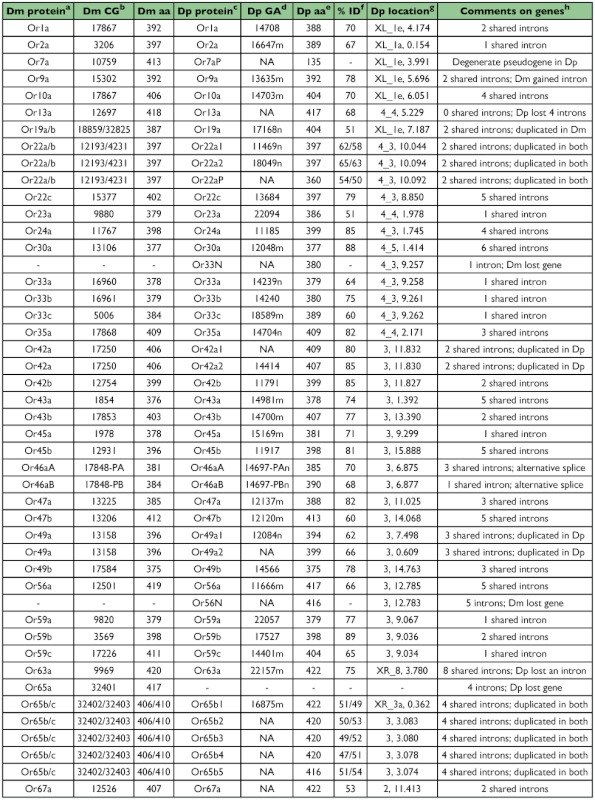
Comparison of Or family genes and proteins in *Drosophila melanogaster* and *D. pseudoobscura*.

**Table:**
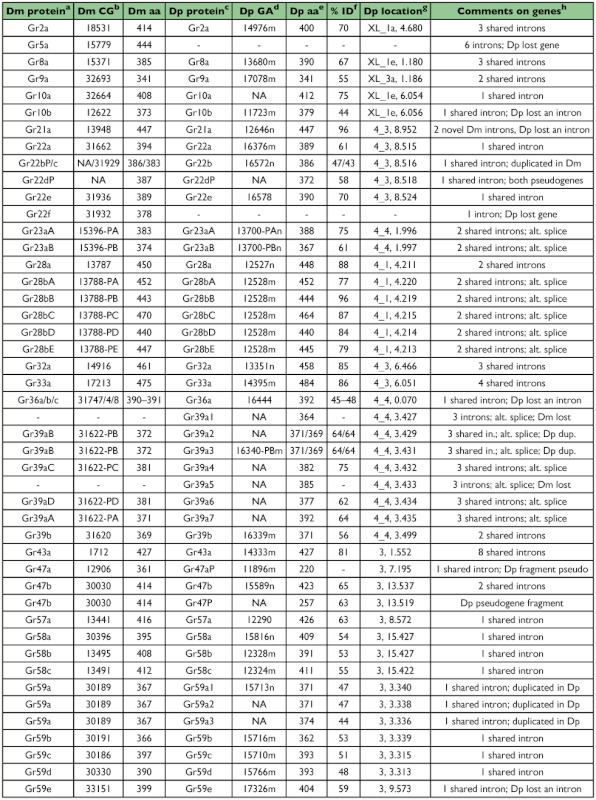
Comparison of Gr family genes and proteins in *Drosophila melanogaster* and *D. pseudoobscura*.

## **Abbreviations**

Or: odorant receptor, a recently expanded family within the insect chemoreceptor superfamily whose members to date have all been shown to function as olfactory receptors, except for Or83b which is co-expressed with other Ors and appears to have a “chaperone” function as a heterodimeric partner with all the specific Ors,

Gr: gustatory receptor, members of the Gr family represent most of the protein diversity in the insect chemoreceptor superfamily, and include both functional gustatory receptors and olfactory receptors, e.g. Gr21a and Gr63a form a heterodimeric receptor for carbon dioxide

## References

[bibr01] Bray S, Amrein H (2003). A putative Drosophila pheromone receptor expressed in male-specific taste neurons is required for efficient courtship.. *Neuron*.

[bibr02] Benton R, Sachse S, Michnick SW, Vosshall LB (2006). Atypical membrane topology and heteromeric function of *Drosophila* odorant receptors in vivo.. *PLoS Biology*.

[bibr03] Chyb S, Dahanukar A, Wickens A, Carlson JR (2003). Drosophila Gr5a encodes a taste receptor tuned to trehalose.. *Proceedings of the National Academy of Sciences USA* 100(Suppl.

[bibr04] Clyne PJ, Warr CG, Freeman MR, Lessing D, Kim J, Carlson JR (1999). A novel family of divergent seven-transmembrane proteins: candidate odorant receptors in *Drosophila*.. *Neuron*.

[bibr05] Clyne PJ, Warr CG, Carlson JR (2000). Candidate taste receptors in *Drosophila*.. *Science*.

[bibr06] Dahanukar A, Lei YT, Kwon JY, Carlson JR (2007). Two Gr genes underlie sugar reception in *Drosophila*.. *Neuron*.

[bibr07] Dekker T, Ibba I, Siju KP, Stensmyr MC, Hansson BS (2006). Olfactory shifts parallel superspecialism for toxic fruit in *Drosophila melanogaster* sibling, *D. sechellia*.. *Current Biology*.

[bibr08] Dobritsa AA, van der Goes van Naters W, Warr CG, Steinbrecht RA, Carlson JR (2003). Integrating the molecular and cellular basis of odor coding in the *Drosophila* antenna.. *Neuron*.

[bibr09] Drosophila 12 Genomes Consortium (2007). Evolution of genes and genomes on the *Drosophila* phylogeny.. *Nature*.

[bibr10] Dunipace L, Meister S, McNealy C, Amrein H (2001). Spatially restricted expression of candidate taste receptors in the *Drosophila* gustatory system.. *Current Biology*.

[bibr11] Ebbs ML, Amrein H (2007). Taste and pheromone perception in the fruit fly *Drosophila melanogaster*.. *Pflugers Archives*.

[bibr12] Ejima A, Smith BP, Lucas C, van der Goes van Naters W, Miller CJ, Carlson JR, Levine JD, Griffith LC (2007). Generalization of courtship learning in *Drosophila* is mediated by cis-vaccenyl acetate.. *Current Biology*.

[bibr13] Engsontia P, Sanderson A, Cobb M, Walden KKO, Robertson HM, Brown S (2008). The red flour beetle's large nose: an expanded odorant receptor gene family in *Tribolium castaneum*.. *Insect Biochemistry and Molecular Biology*.

[bibr14] Guo S, Kim J (2007). Molecular evolution of *Drosophila* odorant receptor genes.. *Molecular Biology and Evolution*.

[bibr15] Ha TS, Smith DP (2006). A pheromone receptor mediates 11-cis-vaccenyl acetate-induced responses in *Drosophil*.. *Journal of Neuroscience*.

[bibr16] Hallem EA, Carlson JR (2006). Coding of odors by a receptor repertoire.. *Cell*.

[bibr17] Hill CA, Fox AN, Pitts RJ, Kent LB, Tan PL, Chrystal MA, Cravchik A, Collins FH, Robertson HM, Zwiebel LJ (2002). G protein-coupled receptors in *Anopheles gambiae*.. *Science*.

[bibr18] Jeanmougin F, Thompson JD, Gouy M, Higgins DG, Gibson TJ (1998). Multiple sequence alignment with Clustal X.. *Trends in Biochemical Sciences*.

[bibr19] Jones WD, Cayirlioglu P, Kadow IG, Vosshall LB (2007). Two chemosensory receptors together mediate carbon dioxide detection in *Drosophila*.. *Nature*.

[bibr20] Kiely A, Authier A, Kralicek AV, Warr CG, Newcomb RD (2007). Functional analysis of a *Drosophila melanogaster* olfactory receptor expressed in Sf9 cells.. *Journal of Neuroscience Methods*.

[bibr21] Krieger J, Grosse-Wilde E, Gohl T, Dewer YM, Raming K, Breer H (2004). Genes encoding candidate pheromone receptors in a moth (*Heliothis virescens*).. *Proceedings of the National Academy of Sciences USA*.

[bibr22] Krieger J, Grosse-Wilde E, Gohl T, Breer H (2005). Candidate pheromone receptors of the silkmoth *Bombyx mori*.. *European Journal of Neuroscience*.

[bibr23] Krieger J, Klink O, Mohl C, Raming K, Breer H (2003). A candidate olfactory receptor subtype highly conserved across different insect orders.. *Journal of Comparative Physiology A Neuroethology and Sensory Neural Behavioral Physiology*.

[bibr24] Kwon JY, Dahanukar A, Weiss LA, Carlson JR (2007). The molecular basis of CO2 reception in *Drosophila*.. *Proceedings of the National Academy of Sciences USA*.

[bibr25] Larsson MC, Domingos AI, Jones WD, Chiappe ME, Amrein H, Vosshall LB (2004). Or83b encodes a broadly expressed odorant receptor essential for *Drosophila* olfaction.. *Neuron*.

[bibr26] Lundin C, Käll L, Kreher SA, Kapp K, Sonnhammer EL, Carlson JR, Heijne G, Nilsson I (2007). Membrane topology of the Drosophila OR83b odorant receptor.. *FEBS Letters*.

[bibr27] Marella S, Fischler W, Kong P, Asgarian S, Rueckert E, Scott K (2006). Imaging taste responses in the fly brain reveals a functional map of taste category and behavior.. *Neuron*.

[bibr28] McBride CS (2007). Rapid evolution of smell and taste receptor genes during host specialization in *Drosophila sechellia*.. *Proceedings of the National Academy of Sciences USA*.

[bibr29] McBride CS, Arguello JR, O'Meara BC (2007). Five *Drosophila* genomes reveal nonneutral evolution and the signature of host specialization in the chemoreceptor superfamily.. *Genetics*.

[bibr30] Neuhaus EM, Gisselmann G, Zhang W, Dooley R, Stortkuhl K, Hatt H. (2005). Odorant receptor heterodimerization in the olfactory system of *Drosophila melanogaster*.. *Nature Neuroscience*.

[bibr31] Nozawa M, Nei M (2007). Evolutionary dynamics of olfactory receptor genes in *Drosophila* species.. *Proceedings of the National Academy of Sciences USA*.

[bibr32] Richards S, Liu Y, Bettencourt BR, Hradecky P, Letovsky S, Nielsen R, Thornton K, Hubisz MJ, Chen R, Meisel RP, Couronne O, Hua S, Smith MA, Zhang P, Liu J, Bussemaker HJ, van Batenburg MF, Howells SL, Scherer SE, Sodergren E, Matthews BB, Crosby MA, Schroeder AJ, Ortiz-Barrientos D, Rives CM, Metzker ML, Muzny DM, Scott G, Steffen D, Wheeler DA, Worley KC, Havlak P, Durbin KJ, Egan A, Gill R, Hume J, Morgan MB, Miner G, Hamilton C, Huang Y, Waldron L, Verduzco D, Clerc-Blankenburg KP, Dubchak I, Noor MA, Anderson W, White KP, Clark AG, Schaeffer SW, Gelbart W, Weinstock GM, Gibbs RA (2005). Comparative genome sequencing of *Drosophila pseudoobscura*: chromosomal, gene, and cis-element evolution.. *Genome Research*.

[bibr33] Robertson HM, Kent LB (2009). Evolution of the gene lineage encoding the carbon dioxide receptor in insects.. *Journal of Insect Science*..

[bibr34] Robertson HM, Wanner KW (2006). The chemoreceptor superfamily in the honey bee, *Apis mellifera*: expansion of the odorant, but not gustatory, receptor family.. *Genome Research*.

[bibr35] Robertson HM, Warr CG, Carlson JR (2003). Molecular evolution of the insect chemoreceptor gene superfamily in *Drosophila melanogaster*.. *Proceedings of the National Academy of Sciences USA* 100(Suppl.

[bibr36] Sakurai T, Nakagawa T, Mitsuno H, Mori H, Endo Y, Tanoue S, Yasukochi Y, Touhara K, Nishioka T (2004). Identification and functional characterization of a sex pheromone receptor in the silkmoth *Bombyx mori*.. *Proceedings of the National Academy of Sciences USA*.

[bibr37] Schmidt HA, Strimmer K, Vingron M, von Haeseler A (2002). TREE-PUZZLE: maximum likelihood phylogenetic analysis using quartets and parallel computing.. *Bioinformatics*.

[bibr38] Scott K, Brady R, Cravchik A, Morozov P, Rzhetsky A, Zuker C, Axel R (2001). A chemosensory gene family encoding candidate gustatory and olfactory receptors in *Drosophila*.. *Cell*.

[bibr39] Slone J, Daniels J, Amrein H (2007). Sugar receptors in *Drosophila*.. *Current Biology*.

[bibr40] Swofford DL (2001). *PAUP**: *Phylogenetic Analysis Using Parsimony and Other Methods*, *Version 4*..

[bibr41] Thorne N, Amrein H (2008). Atypical expression of *Drosophila* gustatory receptor genes in sensory and central neurons.. *The Journal of Comparative Neurology*.

[bibr42] Thorne N, Chromey C, Bray S, Amrein H (2004). Taste perception and coding in *Drosophila*.. *Current Biology*.

[bibr43] Tribolium Genome Sequencing Consortium (2008). The genome of the model beetle and pest *Tribolium castaneum*.. *Nature*.

[bibr44] Tunstall NE, Sirey T, Newcomb RD, Warr CG (2007). Selective pressures on *Drosophila* chemosensory receptor genes.. *Journal of Molecular Evolution*.

[bibr45] van der Goes van Naters W, Carlson JR (2007). Receptors and neurons for fly odors in *Drosophila*.. *Current Biology*.

[bibr46] Vosshall LB, Amrein H, Morozov PS, Rzhetsky A, Axel R (1999). A spatial map of olfactory receptor expression in the *Drosophila* antenna.. *Cell*.

[bibr47] Wanner KW, Anderson AR, Trowell SC, Theilmann DA, Robertson HM, Newcomb RD (2007a). Female-biased expression of odourant receptor genes in the adult antennae of the silkworm, *Bombyx mori*.. *Insect Molecular Biology*.

[bibr48] Wanner KW, Nichols AS, Walden KKO, Brockmann A, Luetje CW, Robertson HM (2007b). A honey bee odorant receptor for the queen substance 9-oxo-2-decenoic acid.. *Proceedings of the National Academy of Sciences USA*.

[bibr49] Wang Z, Singhvi A, Kong P, Scott K (2004). Taste representations in the *Drosophila* brain.. *Cell*.

[bibr50] Wistrand M, Kall L, Sonnhammer EL (2006). A general model of G protein-coupled receptor sequences and its application to detect remote homologs.. *Protein Science*.

[bibr51] Jiao Y, Moon SJ, Montell C (2007). A *Drosophila* gustatory receptor required for the responses to sucrose, glucose, and maltose identified by mRNA tagging.. *Proceedings of the National Academy of Sciences USA*.

